# On Superfœtation

**Published:** 1810-08

**Authors:** 


### *To the Editors of the Medical and Physical Journal.*

GENTLEMEN,

I Am a friend to true and candid criticism, and therefore am obliged to your correspondent A. B. for having offered some remarks upon my letter on Superfœtation; not that I think he has exactly, in all respects, treated it in the manner I could have wished, because the legitimate and *sole* end of criticism is truth; therefore, whenever I find a person turning aside from the points on which the argument depends, to ridicule unimportant deviations from logical precision, I begin to fear, that, in his heart, there is some hankering after victory, to sully the purity of his endeavours in the cause of scientific truth. I need scarcely add my thoughts on the observations of A. B. on the lapsus which which had glided into the first part of my last letter; especially too, in a case like the present, which, when examined with the context, may be defended as being no lapsus at all, because it merely resolves itself into this question, have "the majority of practitioners attentively considered the anatomy of the gravid uterus?" It may be my opinion that they have not.

I consider it extremely unfortunate that I was ignorant of the papers upon the same subject, which appeared in Nos. 125 and 128, of your Journal. From my seeing the Medical Journal only through the uncertain medium of a Book Society, those papers had and still have escaped my notice, so that I am still as ignorant of their contents as I was before the publication of my last letter.*

I now proceed to examine A. B's objections to what he has *made* me, somewhat ostentatiously, give the title of a new doctrine of superfœtation. A. B. does not in any place deny my conclusions, except, indeed, indirectly in one instance, where he shows a considerable want of knowledge in the practice of midwifry,† but levels his shafts at the arguments which are brought forward to prove them; and at these merely upon the principle, that I consider them the *only* cause which prevents superfœtation from taking place. That this is my opinion cannot be deduced from my premises; because, although the road to the ovarium being stopped, may be considered as a cause adequate to the effect, yet, that is no reason why there should not be other causes acting, and acting more forcibly, for the same end. The truth is, that in proving an opinion, you can only make use of tangible arguments. I could say, as well as A. B. or Dr. Haighton, that after impregnation has taken place, the organs of generation become changed in their powers and functions, which disqualifies them from further impregnation as long as gestation is going on; but this is mere opinion; we are unable to demonstrate these changes, and therefore it becomes an inadmissible argument on such an occasion as the present. A. B. indeed remarks, that they are made clear by the experiments of Dr. Haighton.

* In this place A. B. has given an unqualified assertion, as a quotation from my letter, which upon re-perusal he will find is qualified with the word perhaps ———

† He here states, that the plug which stops up the os uteri during gestation might become the cause of rupture of the uterus, when he ought to have known that the first act of labour is the expulsion of this plug.

Haighton. What these experiments are he does not mention; and as I have been unfortunately debarred the opportunity of gaining information from the learning of that gentleman, I should feel much gratified if A. B. would favour me with the proofs upon which he grounds that opinion; for, as circumstances at present strike my mind, there seem to be several facts which are in opposition to it. Perhaps, however, this seeming contradiction may only depend upon the different times at which we fix the termination of impregnation. If Dr. Haighton considers the process of impregnation not to have terminated till the fœtus has descended into the uterus, such an opinion can never be correct, because abundance of cases have happened in which the ovum never has reached the uterus at all, yet still is nourished; but if, on the contrary, he thinks it takes place immediately upon the liberation of the impregnated ovum from the ovarium (and I conceive that no intermediate time can be allowed); then the fact related in my letter from Dr. Hamilton, of bitches bearing puppies of different breed and others of the same kind, seem to stand in direct opposition to the opinion above mentioned. Of this kind, of course, is the fact of the American black woman, mentioned in my letter, which A. B. rather ungraciously doubts being a fact, upon the mere inaccuracy of Mr. Fawell or some other person, who happened to say, that the children were one black and the other *white*, which the commonest understanding must know could not possibly have been the case, as one child must have been a black and the other a *mulatto*. I cannot help finding fault also with A. B. for *making* me say, that, in my opinion, the fright received by the woman was the cause of the death of the child; this he will find was no opinion of *mine* in the slightest degree.

I should like A. B. to be a little more exact in his examination of my expressions, especially after having begun his remarks by finding fault with their accuracy.

I shall with great pleasure see a more satisfactory answer from A. B. for at present I must own, that I do not think he has established any thing of consequence against my positions.

I am, &c.

H.

*July* 8, 1810.

